# Room Temperature Nanoencapsulation of Bioactive Eicosapentaenoic Acid Rich Oil within Whey Protein Microparticles

**DOI:** 10.3390/nano11030575

**Published:** 2021-02-25

**Authors:** Juan David Escobar-García, Cristina Prieto, Maria Pardo-Figuerez, Jose M. Lagaron

**Affiliations:** 1 Research & Development Department, Bioinicia S.L., Calle Algepser 65, 46980 Paterna, Spain; juaesga@gmail.com (J.D.E.-G.); mpardo@iata.csic.es (M.P.-F.); 2 Novel Materials and Nanotechnology Group, Institute of Agrochemistry and Food Technology (IATA), Spanish Council for Scientific Research (CSIC), Calle Catedrático Agustín Escardino Benlloch 7, 46980 Paterna, Spain

**Keywords:** encapsulation, WPC microparticles, EPA-rich oil, electrospraying, oxidative and thermal stability, personalized nutrition

## Abstract

In this study, emulsion electrospraying assisted by pressurized gas (EAPG) has been performed for the first time to entrap ca. 760 nm droplets of the bioactive eicosapentaenoic acid (EPA)-rich oil into whey protein concentrate (WPC) at room temperature. The submicron droplets of EPA oil were encapsulated within WPC spherical microparticles, with sizes around 5 µm. The EPA oil did not oxidize in the course of the encapsulation performed at 25 °C and in the presence of air, as corroborated by the peroxide value measurements. Attenuated Total Reflection—Fourier Transform Infrared spectroscopy and oxygen consumption tests confirmed that the encapsulated EPA-rich oil showed increased oxidative stability in comparison with the free oil during an accelerated oxidation test under ultraviolet light. Moreover, the encapsulated EPA-rich oil showed increased thermal stability in comparison with the free oil, as measured by oxidative thermogravimetric analysis. The encapsulated EPA-rich oil showed a somewhat reduced organoleptic impact in contrast with the neat EPA oil using rehydrated powdered milk as a reference. Finally, the oxidative stability by thermogravimetric analysis and organoleptic impact of mixtures of EPA and docosahexaenoic acid (DHA)-loaded microparticles was also studied, suggesting an overall reduced organoleptic impact compared to pure EPA. The results here suggest that it is possible to encapsulate 80% polyunsaturated fatty acids (PUFAs)-enriched oils by emulsion EAPG technology at room temperature, which could be used to produce personalized nutraceuticals or pharmaceuticals alone or in combination with other microparticles encapsulating different PUFAs to obtain different targeted health and organoleptic benefits.

## 1. Introduction

Understanding the relationship between nutrition and health has resulted in the development of new products; most of them aimed at preventing nutrition-related diseases and improve physical and mental well-being [1]. Polyunsaturated fatty acids (PUFAs) are one of the most studied compounds in healthcare, according to the Global Organization for EPA and DHA Omega-3 (GOED Omega-3) [2]. Hence, there are more than 36,000 published studies on PUFAs, including more than 4000 human clinical trials. Numerous investigations have demonstrated that PUFAs are fundamental for growth and development as well as due to their positive effects on behavior and on the health of the brain, eyes, heart, joints, and skin [3]. Therefore, there is a great interest from the industry in incorporating PUFAs into the active ingredient market. Currently, the PUFAs market is based, especially on alpha-linolenic acid (ALA) C18:3, eicosapentaenoic acid (EPA) C20:5, and docosahexaenoic acid (DHA) C22:6. However, DHA and EPA are claimed to have the most potent health benefits of the PUFAs [4]. Differential biochemical and physiological responses to EPA and DHA have been observed [5]. In particular, EPA is a precursor of multiple metabolites performing essential functions in biological membranes and serves as a precursor for numerous lipid regulators in cellular metabolism [6]. Moreover, it is involved in the therapy of certain illnesses, such as protective effect in liver steatosis [7], correction of postprandial hypertriglyceridemia, hyperglycemia and insulin secretion ability [8], reduction of the occurrence of major coronary event (MCE) [9,10], antiatherosclerotic effects [11], anti-inflammatory effects, depression, hyperactivity disorders [12], and joint health, among others. However, the poor solubility, low stability and limited bioavailability of this sensitive bioactive compound highly reduced its intended health effect, reduced the food product lifetime, and produced consumerist reluctance due to bad organoleptic properties, which greatly limited their application in the health-enhancing products industry.

The main challenge to overcome in the production of functional foods based on omega-3 PUFAs is their oxidation, which produces mainly peroxides, alcohols and aldehydes [13], reducing their nutritional value and increasing their organoleptic impact [2]. Several approaches have been developed to minimize oxidative deterioration and producing stable and easy-to-handle forms [3], improving dispersibility, masking off-flavors and increasing bioavailability [14]. Several strategies employ antioxidants, limit the exposure to ambient air, refine and blanket storage containers with inert gases or make use of microencapsulation. Among them, microencapsulation is one of the most promising approaches, which consists of the entrapment of the bioactive compound within a protective matrix, which restricts the interaction of the functional ingredient with the environment [15]. Moreover, microencapsulation increases bioavailability, reduces organoleptic impact and favors the controlled delivery of these functional ingredients. Microencapsulation processes are classified into physical and chemical methods, being spray-drying, freeze-drying, coacervation and fluid bed coating, the most common methods used until now for the encapsulation of bioactives [16,17]. However, these methods involve temperatures over 100 °C or non-food grade chemicals that could impact the bioactivity of the functional ingredient or prevent its use in food.

Electrospraying is an innovative technology based on electrically charged polymer solution, which after drying, leads to micro- or nanoparticles [15]. Electrospraying is a simple and adaptive technology performed at ambient temperature, which prevents bioactivity loss, and does not need a posterior phase to obtain dry powder [15]. The use of high voltage results in high encapsulation efficiency and controlled particle size distribution. This process presents additional strong points as the large variety of potential carrier matrices, such as carbohydrates, gums, lipids, proteins and waxes, are used either alone or in combination [18,19]. However, the principal disadvantage of the electrospraying technology is its low yield [20], which has restricted the technology transfer to industry. In this sense, Lagaron et al. [21] set up a novel high-yield technology based on the integration of electrospraying and gas-driven nebulization, called electrospraying assisted by pressurized gas (EAPG). In this process, a polymer solution is atomized by means of a pneumatic injector to produce nebulized droplets, which are further size reduced to form encapsulates within a high electric field at the ambient temperature inside a drying compartment, being recovered as a non-agglomerated powder. The advantages of this technique have been previously proven for the encapsulation of fish oil into zein [21,22] and into the combination of whey protein concentrate and carbohydrates [23], and the encapsulation of algae oil into whey protein concentrate and maltodextrin [24]. These works have demonstrated the potential of this technology to stabilize DHA-enriched algae and fish oils. However, as far as we know, no scientific studies have been published dealing with the encapsulation of EPA-rich oils by electrospraying or EAPG.

The main objective of the present study is to investigate the submicron encapsulation of an EPA-rich oil via EAPG technology to produce EPA encapsulates, which could be used in personalized nutrition alone or in combination with other DHA encapsulates. Whey protein concentrate was selected as the wall material because of its amphiphilic, gelation and protective qualities [25]. Produced particles were characterized in relation to their morphologic attributes, oil entrapment efficiency, stability against oxidation, and organoleptic characteristics into a food reference. Finally, thermal stability and organoleptic impact were also studied in combination with DHA encapsulates.

## 2. Materials and Methods

### 2.1. Materials

EPA-rich oil was provided by Solutex GC S.L. (Madrid, Spain) as Omegatex 8000TGD. According to the producer, the EPA content is >800 mg/g as triglycerides. The oil was maintained blanketed with nitrogen, in darkness, at −20 °C. Whey protein concentrate (WPC) 80% was purchased from Beurrespa (Madrid, Spain). According to the distributor, WPC contained 81.6% protein (on a dry basis), 7.5% fat and 4.3% moisture. Algae oil was provided by Q’omer Bioactive Ingredients (Valencia, Spain) with a DHA content of 40 wt %, according to the manufacturer. DHA-loaded WPC microparticles were obtained similarly to the work of Prieto and Lagaron [24]. Span 20 and hydrochloric acid 37 vol % were purchased at Sigma-Aldrich (Saint Louis, MO, USA). Ammonium thiocyanate (99%) and isopropanol (99.5%) were supplied by Acros Organics (Geel, Belgium). Barium chloride dihydrate (reagent grade), iron (III) chloride hexahydrate (PRS), chloroform (99%) and methanol (reagent grade) were obtained from Panreac Química SLU (Barcelona, Spain). Iron (II) sulfate heptahydrate (analytical grade) was supplied by Labkem–Labbox (Mataró, Spain). Isooctane (≥99.0%) was purchased from Honeywell (Morristown, NJ, USA). Bottled mineral water was supplied by Agua de Broncales (Teruel, Spain). Ethanol 96 vol % was obtained from Guinama (Valencia, Spain). Skimmed powdered milk was purchased at Pirinea S.L. (Getafe, Spain). Deionized water was employed during the investigation.

### 2.2. Emulsion Preparation Procedure

An oil in water emulsion was used to encapsulate the EPA-rich oil into WPC. The continuous aqueous phase was elaborated by WPC dissolution into the water at 20% *w/w* concentration. Span 20, which was used as a surfactant, was added to the aqueous solution at 1% *w/w* concentration. EPA-rich oil, which constituted the organic dispersed phase, was gently incorporated to the continuous phase into a 2:1 WPC to EPA ratio. The emulsion was blended with an UltraTurrax T-25 homogenizer (IKA, Staufen, Germany) at 17,000 rpm for 5 min, succeeded by 5 min of ultrasounds (90%) (Bandelin Sonopuls, Berlin, Germany). The emulsion was placed in chilled water to prevent the temperature from rising, and constant nitrogen bubbling was maintained to avoid EPA oxidation during homogenization. Particles obtained from a WPC solution without EPA oil were used as a control sample.

### 2.3. Emulsion Droplet Size

Laser diffraction analysis was performed in a Mastersizer 2000 (Malvern Instruments, Ltd., Worcestershire, UK) to measure the emulsion droplet size. A small volume of the emulsion was introduced in recirculating water at 3000 rpm to obtain an obscuration of 12%. The refractive indices for the dispersed phase and the continuous phase were that of sunflower oil (1.469) and water (1.330), respectively. Results were expressed in terms of the Sauter mean diameter (D_3,2_). All measurements were performed three times.

### 2.4. Encapsulation Process

EPA encapsulation was performed by EAPG by means of the proprietary Capsultek^TM^ pilot plant from Bioinicia S.L. (Valencia, Spain). This pilot device is composed of a nebulizer, the droplets product of which are subjected to an electric field, an evaporating compartment, and a cyclone as described by Busolo et al. [21]. All tests were developed at controlled ambient conditions, 25 °C and 30% relative humidity (RH). EPA-rich oil emulsion was exposed to a continuous bubbling of nitrogen, and it was pumped at a flow rate of 30 mL/h to the injection unit, working with an airflow rate of 10 L/min and a voltage of 10 kV. EPA-loaded WPC capsules were recovered from the cyclonic unit and kept in airtight containers, in the dark, at −20 °C to prevent oxidation.

### 2.5. Particle Morphology Analysis

Scanning electron microscopy (SEM) was used to study the morphology of the particles in a Hitachi S-4800 FE-SEM (Hitachi High Technologies Corp., Tokyo, Japan). An acceleration of 5 kV for the electron beam was selected. Around 5 mg of each sample was sputtered with gold/palladium before SEM observation. Average diameters were obtained via the software Image J Launcher v1.41 (National Institutes of Health, Bethesda, MD, USA).

Transmission electron microscopy (TEM) was employed to examine the internal morphology of the particles in a JEOL JEM 1010 (JEOL Ltd., Tokyo, Japan). Particles were subjected to an inclusion process in LR-white resin and subsequent polymerization. Ultrathin sections were cut by means of an ultramicrotome and placed on the TEM grid [26].

### 2.6. Evaluation of the Extractable Oil

Extractable EPA-rich oil (EO) from the particles was quantified using UV-vis spectrophotometry. 25 mg of EPA–loaded WPC capsules were thoroughly washed with 5 mL of isooctane, and the mixture was filtrated. The absorbance of the filtrated liquid phase was determined at 318 nm using a UV4000 spectrophotometer (Dinko Instruments, Barcelona, Spain). Standard curve of EPA-rich oil in isooctane was built from 0.0 to 1.0 mg/mL (*y* = 0.0217*x* + 0.0015; *R*^2^ = 0.98) to calculate the amount of EPA-rich oil solubilized in the isooctane. The extractable oil was calculated as the percentage of the extracted EPA-rich oil with respect to the total mass of EPA-rich oil in the particle. The procedure was performed three times.

### 2.7. Accelerated Stability Test

A 300 W Ultra-Vitalux lamp from OSRAM (Garching, Germany) was employed to irradiate samples with ultraviolet (UV) light and accelerate the oxidation of EPA-rich oil. This lamp made up of a quartz discharge tube and a tungsten filament, generates radiation analogous to that of daylight. The bulb, made of special glass, filters radiation and allows only wavelengths similar to natural sunlight to pass through (315–400 nm, 13.6 W after 1 h of exposure; 280–315 nm, 3 W after 1 h of exposure) [27,28].

The accelerated oxidative stability test was performed at room conditions under UV light for 10 days. Approximately, Petri dishes with 10 g of particles were located under a UV lamp. Daily, aliquots were sampled for analysis. The distance between the lamp and the samples were 20 cm. The evolution of the oxidation was measured by attenuated total reflection- Fourier-transform infrared spectroscopy and peroxide value.

### 2.8. Peroxide Value (PV) Evaluation

The peroxide value determination was performed according to the methodology described elsewhere [24]. The oil was extracted from the particles according to the Bligh and Dyer method [29], and it was analyzed according to the PV method of Shantha and Decker [30]. PV represented in milliequivalents of peroxides per kilogram of oil was calculated according to Equation (1):

(1) $$PV=\left[\frac{As-Ab}{m}\right]\cdot \left[\frac{V}{\left(2\cdot 55.84\cdot m_{0}\cdot S\right)}\right]$$

where *As* is the absorbance of the test sample and *Ab* is absorbance of blank; *m* is the slope of the standard curve, *m*_0_ is the mass of EPA oil, 55.84 g/mol is the iron atomic weight, *S* is the volume of the oil solution sample, *V* is the volume of solvent employed to solubilize the EPA oil. PV determination was performed three times.

### 2.9. Attenuated Total Reflection—Fourier Transform Infrared Spectroscopy (ATR-FTIR)

A Bruker Tensor 37 FT-IR spectrometer (Bruker, Ettlingen, Germany) with the low-temperature Golden Gate ATR sampling accessory (Specac Ltd., Orpington, UK) was used to obtain the ATR-FTIR spectra of approximately 50 mg EPA-loaded WPC capsules. Spectra were recorded between 4000 and 600 cm^−1^, with a 4 cm^−1^ resolution, averaging 10 scans. Results were normalized to the intensity of the band 1455 cm^−1^ for a better comparison among the different capsules. The OPUS 4.0 software (Bruker, Ettlingen, Germany) was used to perform the analysis of the spectra. Measurements were performed in triplicate.

### 2.10. Headspace Oxygen Volume Depletion

A comparison of the headspace oxygen volume depletion between the free EPA-rich oil and the obtained EPA-loaded WPC capsules was performed using a multichannel oxygen meter OXY-4 mini (PreSens Precision Sensing GmbH, Regensburg, Germany). The analysis over time was carried out via the fluorescence decay method using 2.5 g of EPA-loaded WPC capsules inserted in 100 mL Schleck flasks with spot sensors, at 25 °C and 0% RH. An equivalent amount of EPA-rich oil was used in the case of free EPA-rich oil. Values were taken for 165 h and normalized to the initial headspace oxygen volume. The measurements were performed in duplicate.

### 2.11. Thermogravimetric Analysis (TGA)

Thermogravimetric analysis of encapsulates was performed on a 550-TA Instruments thermogravimetric analyzer (New Castle, DE, USA). Approximately 5 mg of samples were placed on the platinum pan and kept under air atmosphere with a flow rate of 50 mL/min and heated in the temperature range of 25–700 °C, at a heating rate of 10 °C/min.

### 2.12. Organoleptic Test

The organoleptic impact of free EPA-rich oil was compared to the impact of EPA-rich oil-loaded WPC capsules when added to the rehydrated powdered milk, used as a reference. 37.5 mg of EPA-rich oil was added to 25 g of skim milk powder and 130 mL of bottled mineral water. In the case of EPA-rich oil-loaded WPC capsules, 75 mg were added using the mentioned preparation conditions. Eight trained panelists from the IATA-CSIC compared the general fishiness characteristics, including color, odor, taste and appearance, with the model food product (rehydrated milk powder free of oil or capsules). Samples were scored according to a five-point hedonic scale: (0) no difference; (1) little difference; (3) clear difference; and (5) big difference.

A similar analysis was performed comparing the organoleptic impact of the free oil with the encapsulates, but in this case, mixing EPA and DHA enriched oils in a ratio of 50:50 in weight.

### 2.13. Statistical Analysis

Statgraphics Centurion Version 17.2.04 (Statistical Graphics Corp., Warrenton, VA, USA) was used for statistical analysis. Data were represented as mean ± standard deviation. Initially, data were subjected to analysis of variance (ANOVA) to evaluate the effects of the examined parameters on the different samples. Kruskal–Wallis test by ranks was developed to contrast the action of the medians instead of the median values for organoleptic analyses. Differences between means and medians were contemplated as significant at *p*-value < 0.05.

## 3. Results and Discussion

The aim of this research was to study the encapsulation of nanodroplets of EPA-rich oil into WPC microparticles via the emulsion EAPG process. Obtained microcapsules were characterized in terms of morphology, extractable oil, oxidative and thermal stability and organoleptic impact.

### 3.1. Morphology

**Figure 1:**
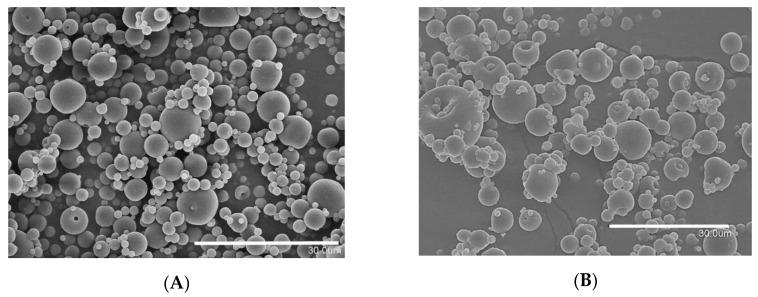
SEM micrographs: (**A**) whey protein concentrate (WPC) microparticles; (**B**) eicosapentaenoic acid (EPA)-rich oil-loaded WPC capsules. Scale bar corresponds to 30 µm.

Neat WPC capsules with an average particle size of 5.11 ± 2.25 µm, spherical structure, with unwrinkled surface and without fissures or dents were obtained, as shown in Figure 1A. The encapsulation of EPA-rich oil into WPC at ratio 2:1 led to a similar microcapsule morphology of neat WPC but with an average particle size of 5.59 ± 2.68 µm (Figure 1B). These kinds of morphological attributes, spherical shape and smooth surface, are suitable for food applications due to a better dispersibility into the final formulation and a minimum texture impact [31]. Particles with similar morphologies were obtained by Prieto and Lagaron when encapsulating algae oil into WPC via EAPG [24] and by García-Moreno et al. when encapsulating fish oil into WPC and carbohydrates by using the same technology [23]. A standard deviation of approximately 2 µm was observed for neat and EPA-loaded microparticles; however, this polydispersion should not affect the organoleptic properties of the final product since the size is far below the tongue detection limit of 20 µm [32].

**Figure 2:**
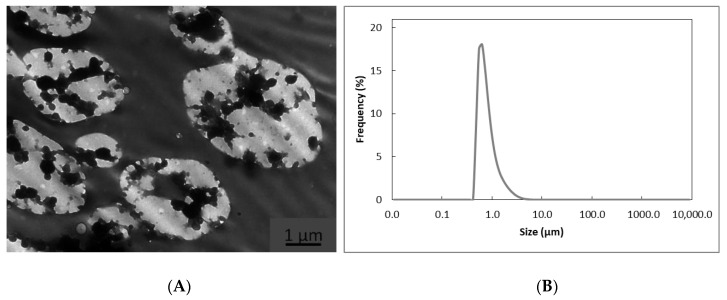
(**A**) TEM image of the EPA-rich oil-loaded WPC capsules. (**B**) Emulsion droplet size distribution obtained by laser diffraction analysis.

Figure 2A shows the manner in which EPA-rich oil was entrapped within the WPC matrix. It consisted of a spongelike structure, which is known to enhance oil preservation [33] and favor the oil absorption in the gastrointestinal tract [34]. The internal morphology of the microcapsules coincides with the measured size, i.e., 0.762 ± 0.001 µm, of the droplets in the emulsion (Figure 2B). The polydispersion observed in the internal nanostructure of the produced microparticles could be due to the lack of stability of the emulsion, which could be optimized to increase the oil stability.

### 3.2. Extractable Oil

UV-vis spectrophotometry was used to measure the extractable EPA-rich oil in the capsules using a thorough oil extraction method. The extractable oil of the here-prepared EPA-rich oil-loaded WPC capsules was 30.0 ± 1.1%. Previous studies of encapsulation of omega-3-rich oils via electrospraying-based technologies reported similar results. Prieto and Lagaron reported an extractable oil in organic solvent of 35 ± 5% for DHA-enriched algae oil encapsulated within WPC using EAPG as encapsulating technology [24]. Gómez-Mascaraque et al. prepared different protein particles for the encapsulation of α-linolenic acid (ALA), finding that the higher oil retention capacity was achieved using WPC and soy protein isolate complex with values of 67 ± 5% and 61 ± 4%, respectively via electrospraying [15]. The increased oil retention obtained for EPA could be due to the inherent characteristics of oil, such as increased lipophilicity or viscosity. Regarding other technologies, Olloqui et al. encapsulated EPA and DHA into calcium alginate, obtaining oil retentions up to 93.5 ± 2.2%. However, this result was obtained with an EPA oil loading of 1% in the alginate beads [35], whereas in this work, the oil loading in the microparticle was 33%.

### 3.3. Accelerated Stability Test

The stability against oxidation of EPA-loaded WPC microparticles versus the free EPA oil was compared by irradiating the samples with UV light for 10 days. PV and ATR-FTIR spectroscopy were used to quantify the EPA-rich oil oxidation.

The PV quantifies the formation of primary fatty acid oxidation products, specifically hydroperoxides. The initial PV for non-encapsulated and encapsulated EPA-rich oil reached 12.7 ± 2.0 meq/kg and 13.8 ± 1.4 meq/kg, respectively. According to the provider, this EPA-rich oil was deodorized, and consequently, it does not surprise the high initial PV value. Nevertheless, the slight difference between the free EPA-rich oil and the encapsulated in WPC could be due to the emulsion preparation and also to the contact of the non-encapsulated oil with oxygen in the course of the EAPG process.

**Figure 3:**
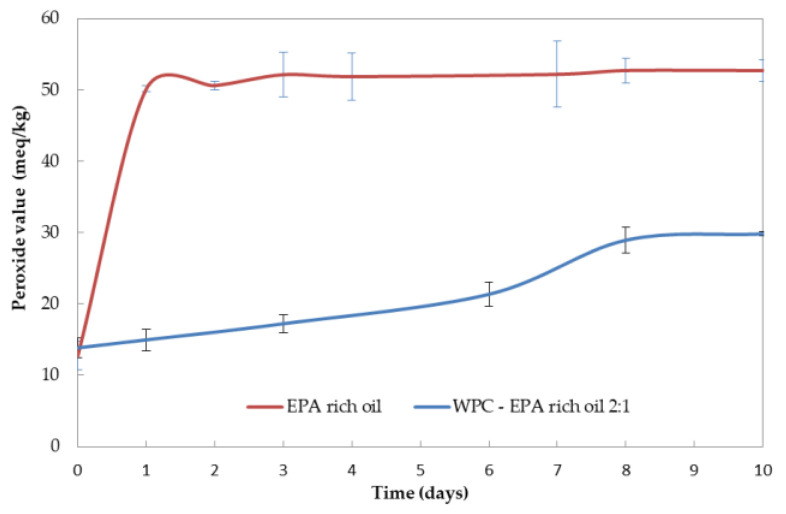
Evolution of the peroxide value of non-encapsulated EPA-rich oil compared with the EPA-loaded WPC microparticles.

Regarding the evolution of the PV with the exposure of the particles to UV light, the PV of the free EPA-rich oil increased rapidly during the first day due to the formation of hydroperoxides, as shown in Figure 3. During the second day, the concentration of hydroperoxides slightly diminished as a consequence of their secondary oxidative conversion into aldehydes, ketones, and alcohols responsible for the off-flavors and consumer reluctance. After the second day, the peroxide value was arrested for the free EPA oil. However, in the case of encapsulated EPA-rich oil into WPC, a slow monotonic increase was obtained. Both curves showed a sigmoid behavior, characteristic of autocatalytic oxidation processes that occur as a chain reaction of free radicals [36]. Lipid oxidation reactions are characterized by accelerating with time, with the possibility of showing an induction period. However, the height of the peroxide value, the general curve shape, and the induction period depends on the oxidation kinetic parameters, as well as the composition of the sample [37]. Thus, the WPC-EPA curve showed a longer induction time. This last behavior could be a consequence of the inherent antioxidant properties of the WPC [38], which could delay the generation of free radicals.

**Figure 4:**
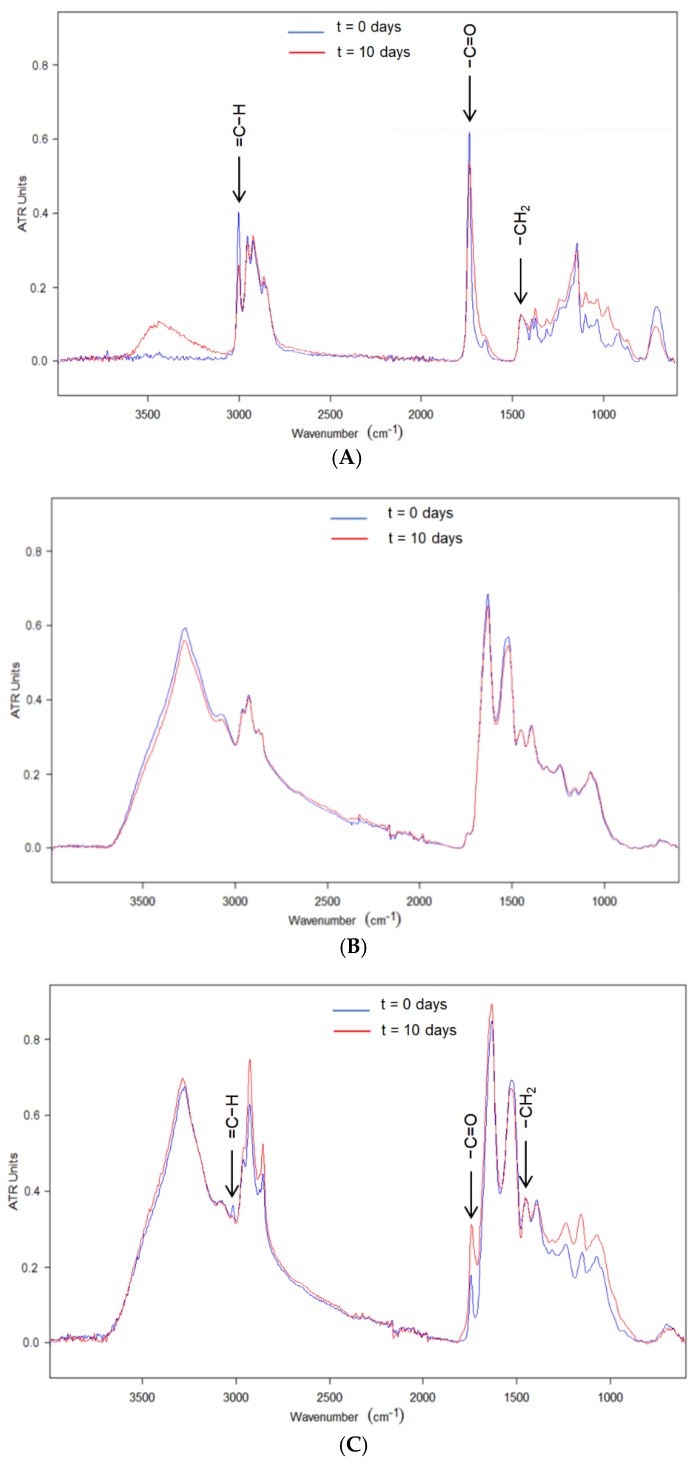
Evolution of the attenuated total reflection—Fourier transform infrared spectroscopy (ATR-FTIR) spectra during the accelerated oxidation test: (**A**) EPA-rich oil, (**B**) WPC and (**C**) WPC-EPA-rich oil microparticles.

ATR-FTIR spectroscopy has been demonstrated to be a good methodology to evaluate the extension of secondary oil oxidation reactions [24]. Initially, the ATR-FTIR spectra of the neat EPA-rich oil were studied in the course of the accelerated oxidative reactions under UV light. The ATR-FTIR spectrum of the fresh non-encapsulated EPA oil is compared in Figure 4A with the ATR-FTIR spectrum of non-encapsulated oxidized EPA oil subjected to 10 days of accelerated oxidation. Spectra were normalized in intensity to ease the comparison. The peak at ca. 1455 cm^−1^ was used as an internal standard for normalization, similarly to the previous study [24]. This band is assigned to the bending vibrations of CH_2_, CH_3_ aliphatic groups [39], which did not show any variation during oxidation. Figure 4A shows the ATR-FTIR spectra of the EPA-rich oil, where main bands were easily identified. The peak at 3012 cm^−1^ corresponds to the stretching vibrations of alkenes in PUFAs. A reduction in the intensity of this band was observed over UV exposure time as a result of the oxidation reactions, in which the desaturations disappear [40,41]. Further overlapping stretching modes from EPA-rich oil appear at 2964, 2919 and 2872 cm^−1^, corresponding to the asymmetric CH_3_, antisymmetric CH_2_ and the symmetric CH_2_ stretching, respectively [42,43]. The higher intensity of the band at 3012 cm^−1^ with respect to the bands at 2964, 2919 and 2872 cm^−1^ confirms the high omega-3 PUFAs content [44] of 80%, according to the producer. It is also remarkable the peak at ca. 1748 cm^−1^ attributed to the ester and acid groups stretching vibrations of triacylglycerols [40,45]. The intensity of this band was reduced at the same time that the band expanded towards lower wavenumbers as a consequence of the formation of primary and secondary oxidation products [39]. The band at 1650 cm^−1^ was assigned to stretching vibrations of C=C bonds, and alterations in intensity suggested the generation of trans double bonds due to radiation [46]. The band at ca. 1145 cm^−1^ has been associated with the number of saturated acyl groups in the oil. The location of this band moved towards larger wavenumbers as a consequence of the oxidation reactions, in which smaller saturated acyl molecules are formed [39]. The peak at ca. 705 cm^−1^, assigned to the convergence of the methylene rocking vibration and the out-of-plane bending vibration of disubstituted cis-alkenes [39], presented a decrease in intensity as oxidative reactions occurred [46].

Regarding WPC, the characteristic bands of proteins at 1650 cm^−1^ and 1550 cm^−1^ attributed to amide I and amide II [47] were easily discerned in Figure 4B. However, these spectra did not show significant alterations as a result of the exposure to UV light for 10 days, only a slight decrease in the intensity of the bands attributed to water, probably due to the warming provoked by the UV light bulb.

**Figure 5:**
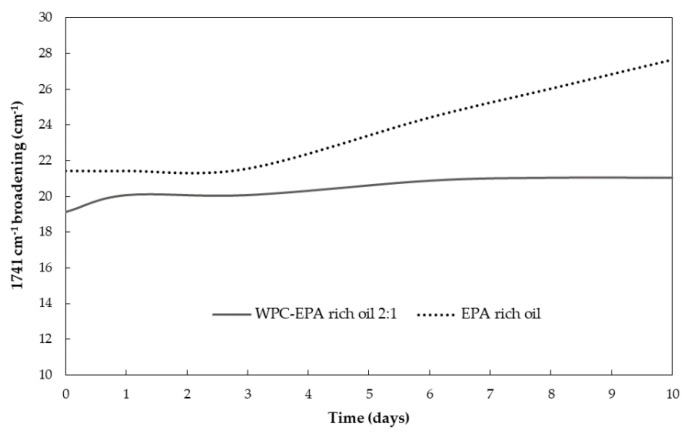
Peak widening of the free EPA-rich oil and the encapsulated EPA-rich oil into WPC, obtained as the 1741 cm^−1^ broadband at half maximum, over UV-light exposure time.

The evolution of the characteristic bands of the EPA-loaded WPC microparticles over UV light exposure time is shown in Figure 4C. The most significant changes were a decrease in the intensity of the band at 3012 cm^−1^ and an apparent increase in the relative intensity of the band at 1741 cm^−1^ in addition to a band broadening of the same band. Via UV-vis spectrophotometry, it was also observed the disappearance of the band at 318 nm due to the oxidation of the oil (results not shown). However, as demonstrated in previous work [24], the widening of the peak at 1741 cm^−1^ is the most suitable to quantify the generation of secondary oxidation products. Therefore, Figure 5 shows the evolution of the 1741 cm^−1^ broadband at half maximum over UV light exposure time. The band broadening of the free EPA-rich oil was maintained stable until the third day, after which it experienced a significant and strong rise in slope. However, the encapsulated EPA-rich oil into WPC showed a moderate and non-significant broadening during the first day, after which it was arrested. EPA-rich oil seemed to be more stable to UV light when compared to DHA enriched oil, which showed a broadening of approximately 40 cm^−1^ after being subjected to 10 days under UV light [24]. The different behavior could be due to the different compositions of both oils. Regarding the encapsulates, DHA-loaded WPC microparticles also showed a non-significant broadening of approximately 6 cm^−1^ after being subjected to 10 days of UV light [24]. The obtained results for the encapsulates indicated that the encapsulation within the WPC protein seems to block UV radiation, decreasing the generation of secondary oxidation products [48].

### 3.4. Headspace Oxygen Volume Depletion Test

**Figure 6:**
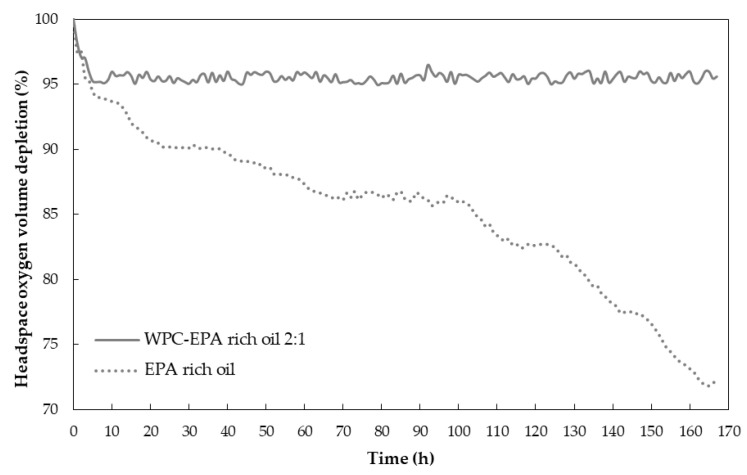
Comparison of the averaged headspace oxygen volume depletion between the neat oil and EPA-loaded WPC microparticles.

The headspace oxygen volume depletion test was performed to study the oxygen barrier capacity of the WPC to protect the EPA-rich oil by the fluorescence decay method at room temperature and 0% RH for an equivalent amount of EPA-rich oil. In the result of the EPA-loaded microparticles, the signal of the neat WPC was subtracted to evaluate only the oxygen depletion due to the oil. As shown in Figure 6, free EPA-rich oil showed a higher oxygen consumption than the encapsulated oil, which implied a higher oxidation rate. The fast oxygen consumption observed at the beginning maybe due to the oxidative reactions of the non-encapsulated oil in the microparticles. After 165 h of experiment, the neat oil showed approximately 28% of oxygen consumption, while the microparticles consumed around 5%. Prieto and Lagaron obtained a 7% of oxygen consumption for the encapsulation of algae oil into WPC using the EAPG technology, whereas free algae oil consumed around 20% [24], and Busolo et al. obtained an 8% of oxygen consumption when encapsulating fish oil into zein using the same technology, whereas the free fish oil consumed around 28% [21]. The obtained results are probably due to the exceptional oxygen-barrier properties of the whey protein concentrate at low relative humidity [49].

### 3.5. Thermogravimetric Analysis

**Figure 7:**
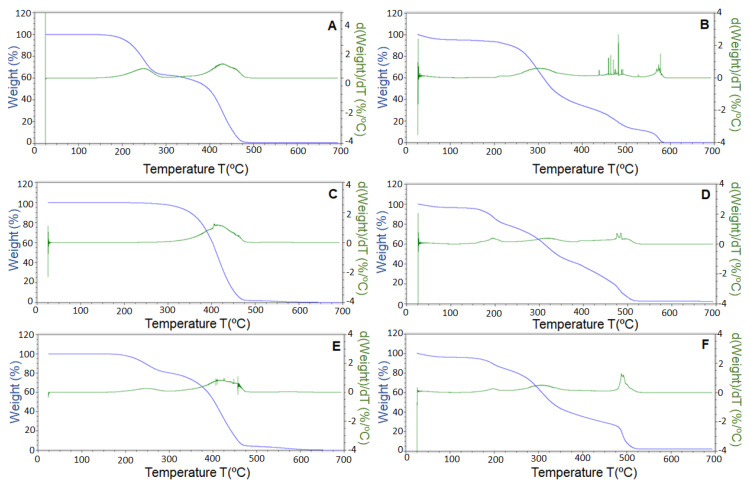
Thermogravimetric analysis curves in the presence of oxygen of (**A**) EPA-rich oil, (**B**) EPA-loaded WPC microparticles, (**C**) docosahexaenoic acid (DHA)-rich oil, (**D**) DHA-loaded WPC microparticles, (**E**) EPA–DHA oils mixture (50:50), (**F**) mixture of EPA-loaded WPC microparticles and DHA-loaded WPC microparticles in mass ratio 50:50.

Thermogravimetric analysis was used to study the thermal stability in the presence of oxygen. This analysis was performed to compare the stability of the free EPA-rich oil and the encapsulated EPA-rich oil. Results are shown in Figure 7A,B. EPA-rich oil demonstrated to be stable until 150 °C, showing a first weight loss of 38% between 150 and 300 °C, followed by a second weight loss of 62% until 490 °C. However, the EPA-loaded WPC microparticles showed increased thermal stability. The microparticles showed a small event under 100 °C attributed to the evaporation of sorbed water on the microparticles, being stable until 200 °C, where microparticles showed a weight loss of 52% until 350 °C, a second weight loss of 27% until 500 °C, and a final weight loss of 14% until 585 °C. This delay in the TGA curve is ascribed to the protection of the WPC.

Since most of the published studies were developed with fish or algae oils rich in DHA, free and encapsulated DHA enriched oil from algae were also studied in order to establish a comparison. Free DHA-enriched oil showed increased thermal stability in comparison with EPA-rich oil since it was stable until 215 °C, as shown in Figure 7C. DHA enriched oil showed a total weight loss between 215 and 600 °C. However, the DHA-loaded microparticles showed (see Figure 7D) a small thermal event due to the humidity sorbed in the microparticles, but a first weight loss of 15% occurred at 110 °C. This was not seen in the pure oil and was probably due to the potential interaction of the oil with the polymer.

Finally, a mixture of EPA and DHA-rich oils was also studied; since both compounds have differential health benefits, EPA and DHA encapsulates could be combined in different ratios with different health effects for personalized medicine or nutrition purposes. Free EPA and DHA oils were combined in a ratio of 50:50, as well as their microparticles. Results are shown in Figure 7E,F, respectively. The combination of EPA and DHA resulted in increased thermal stability in comparison with the neat EPA oil, since the first thermal event occurred at 170 °C, showing a TGA curve similar to that of EPA-rich oil, but with a decreased first thermal event. Regarding the encapsulates, the mixture of microparticles also resulted in an earlier thermal event, showing a similar TGA curve but with a somewhat decreased thermal stability in comparison with the EPA encapsulates.

### 3.6. Organoleptic Properties

**Figure 8:**
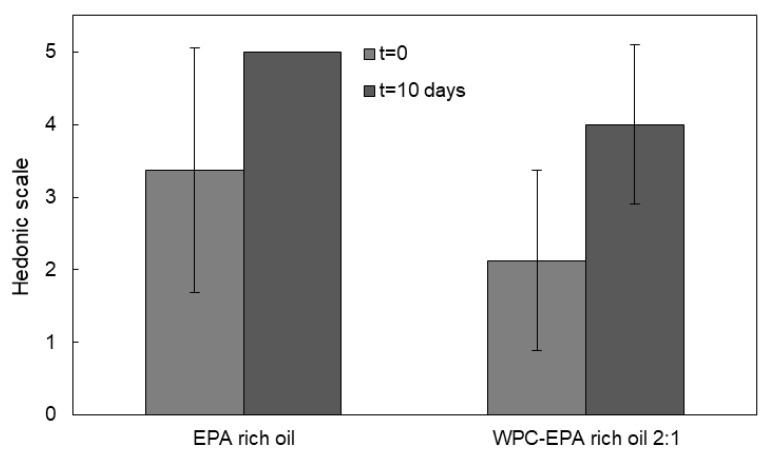
Comparison of the organoleptic impact of reconstituted powder milk containing free oil and EPA-rich oil-loaded microcapsules for fresh samples (t = 0) and upon the completion of the accelerated oxidation test. Data were expressed as mean value ± standard deviation.

The organoleptic impact of the oil-loaded microparticles was evaluated in comparison with the neat EPA-rich oil, employing rehydrated powdered milk as reference. The assay was carried out with fresh samples and the samples upon completion of the accelerated oxidation test. Qualified panelists detected a high organoleptic impact for the free EPA-rich oil, as shown in Figure 8. This result is consistent with the high initial peroxide value and with the fact that the oil suffered from a deodorization process, according to the manufacturer. Nevertheless, the capsules offered a somewhat reduced impact also after 10 days of UV light exposure, according to the panelists, even if the effect was not significant.

**Figure 9:**
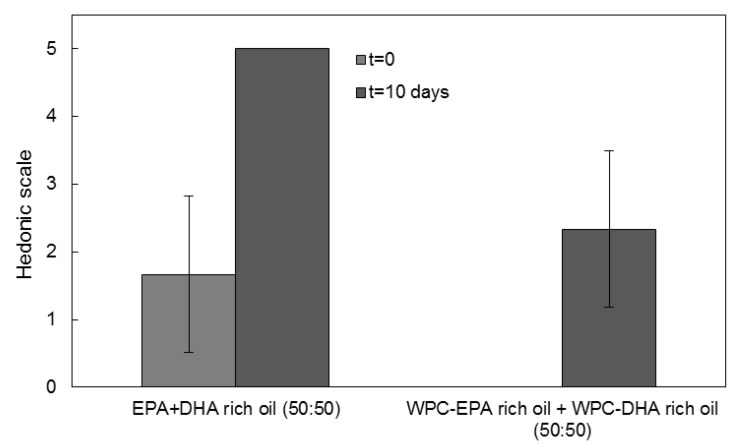
Comparison of the organoleptic impact of reconstituted powder milk containing EPA and DHA enriched free oil (50:50) and EPA and DHA-loaded microcapsules in ratio 50:50, for fresh samples (t = 0) and upon the completion of the accelerated oxidation test. Data were expressed as mean values ± standard deviation.

EPA and DHA encapsulates are also combined in different ratios with different health or organoleptic purposes. In Figure 9, a comparison of the organoleptic impact of the combination of the free mixture of EPA and DHA in weight ratio 50:50 and a mixture of encapsulates of EPA and DHA in a weight ratio of 50:50 is shown. When combining EPA and DHA, the organoleptic impact of the free oil was reduced in comparison to Figure 8 at t = 0, being more similar to the organoleptic impact reported by Prieto and Lagaron [24]. However, the organoleptic impact of the free oil mixture was seen to increase to its maximum after 10 days of UV exposure. The panelist did not report organoleptic impact for the fresh encapsulates, but they reported a reduced organoleptic impact after 10 days of UV exposure in comparison with the pure EPA encapsulates. This is likely related to the lower organoleptic impact of the algae-derived DHA oil. Therefore, the combination of different encapsulated PUFAs can be used to tailor a new line of personalized products with targeted health and organoleptic benefits.

## 4. Conclusions

In this work, EPA-rich oil was first successfully encapsulated for the first time in submicron droplets within whey protein concentrate microparticles using the emulsion electrospraying assisted by the pressurized gas process. The obtained results suggest that it is possible to encapsulate 80% PUFAs-enriched oils by emulsion EAPG technology at room temperature without affecting the bioactivity of the EPA oil into nanostructured microparticles, which favor oil protection and gastric absorption, with an increased oxidative and thermal stability, and a reduced organoleptic impact. Another advantage of this encapsulation process is that it is available at an industrial scale. Furthermore, thanks to the differential health effects of the EPA, these encapsulates could be used in personalized medicines or nutraceuticals, alone or in combination with other microparticles encapsulating PUFAs to obtain targeted health and organoleptic benefits.
